# Discovering the DNA-Binding Consensus of the *Thermus thermophilus* HB8 Transcriptional Regulator TTHA1359

**DOI:** 10.3390/ijms221810042

**Published:** 2021-09-17

**Authors:** Josiah L. Teague, John K. Barrows, Cynthia A. Baafi, Michael W. Van Dyke

**Affiliations:** Department of Chemistry and Biochemistry, Kennesaw State University, Kennesaw, GA 30144, USA; Josiah.Teague@stjude.org (J.L.T.); jbarro51@kennesaw.edu (J.K.B.); cynbaafi@gmail.com (C.A.B.)

**Keywords:** bioinformatics, biolayer interferometry (BLI), electrophoretic mobility shift assay (EMSA), extremophile, protein-DNA binding, type IIS restriction endonuclease

## Abstract

Transcription regulatory proteins, also known as transcription factors, function as molecular switches modulating the first step in gene expression, transcription initiation. Cyclic-AMP receptor proteins (CRPs) and fumarate and nitrate reduction regulators (FNRs) compose the CRP/FNR superfamily of transcription factors, regulating gene expression in response to a spectrum of stimuli. In the present work, a reverse-genetic methodology was applied to the study of TTHA1359, one of four CRP/FNR superfamily transcription factors in the model organism *Thermus thermophilus* HB8. Restriction Endonuclease Protection, Selection, and Amplification (REPSA) followed by next-generation sequencing techniques and bioinformatic motif discovery allowed identification of a DNA-binding consensus for TTHA1359, 5′–AWTGTRA(N)_6_TYACAWT–3′, which TTHA1359 binds to with high affinity. By bioinformatically mapping the consensus to the *T. thermophilus* HB8 genome, several potential regulatory TTHA1359-binding sites were identified and validated in vitro. The findings contribute to the knowledge of TTHA1359 regulatory activity within *T. thermophilus* HB8 and demonstrate the effectiveness of a reverse-genetic methodology in the study of putative transcription factors.

## 1. Introduction

In bacteria, transcription regulatory proteins or transcription factors function as critical constituents of signal transduction networks, acting upon environmental and cellular cues to modulate the transcriptional program appropriately through their specific binding to control elements within targeted gene promoters [1]. While their functions are traditionally determined through genetic means, this is less feasible in many less-well-studied organisms, often relying on genomic organization and structural homology to infer putative transcription factor biological roles [2].

The cyclic-AMP receptor proteins (CRPs) and fumarate and nitrate reduction regulators (FNRs) compose the CRP/FNR superfamily of transcriptional regulators, a diverse subgroup of bacterial transcription factors regulating gene expression in response to a spectrum of stimuli [3]. Currently, insight into the CRP/FNR superfamily primarily derives from past and present research into the founding and representative members of the superfamily, *E. coli* CRP (CRP_Ec_) and FNR (FNR_Ec_), respectively [4,5]. Following complexation with the metabolite effector 3′−5′ cAMP, CRP_Ec_ homodimers adopt a conformation that allows them to bind to DNA sequences with the consensus 5′-AAATGTGAtctagaTCACATTT-3′, thereby regulating hundreds of genes involved with the catabolism of secondary carbon sources [6,7,8]. FNR_Ec_, on the other hand, forms homodimers containing two [4Fe-4S] clusters under anaerobic conditions [9], allowing them to bind DNA sequences having the consensus 5′-TTGATnnnnATCAA-3′ and activating the expression of hundreds of genes involved with anaerobic respiration [10,11,12].

*Thermus thermophilus* HB8 [13] is a model extreme thermophile and is the subject of the Structural-Biological Whole Cell Project, which seeks to understand all cellular biological phenomena at an atomic level [14,15]. High-resolution, three-dimensional structures have been obtained for hundreds of its proteins, owing to their ease in crystallization and x-ray diffraction analysis. The *T. thermophilus* HB8 genome has been fully sequenced [16], and through homology studies, four CRP/FNR transcription factors have been identified: TTHA1359, TTHA1437, and TTHA1567, encoded by genes in the main chromosome, and TTHB099, encoded by a gene in the maxiplasmid pTT27. Structural information has been obtained for three *T. thermophilus* HB8 proteins, TTHA1359, TTHA1437, and TTHB099, with only TTHA1437 requiring cAMP binding to adopt a conformation conducive for DNA binding [17,18,19]. Additionally, information regarding DNA sequences recognized by these transcription factors and their regulated genes has been published for TTHA1359 and TTHA1437, thereby providing insights into their potential biological functions [17,18,20].

Our laboratory has pioneered a reverse-genetic approach to obtain insights into transcription factors in *T. thermophilus* HB8 [21,22,23,24,25]. Such approach entails the iterative selection method Restriction Endonuclease Selection, Protection, and Amplification (REPSA) to define their consensus DNA-binding sequences and various bioinformatic methods to identify favored genomic binding sites, promoter element homologies, and potential biological roles. Here we describe our investigation of the *T. thermophilus* HB8 transcription factor TTHA1359, a CRP/FNR superfamily protein, also known as SdrP [18,20]. We found that TTHA1359 preferentially binds a consensus sequence 5′–AWTGTRA(N)_6_TYACAWT–3′ and identified several genes potentially regulated by this protein.

## 2. Results

### 2.1. REPSA Selection of TTHA1359-Binding Sequences

**Figure 1:**
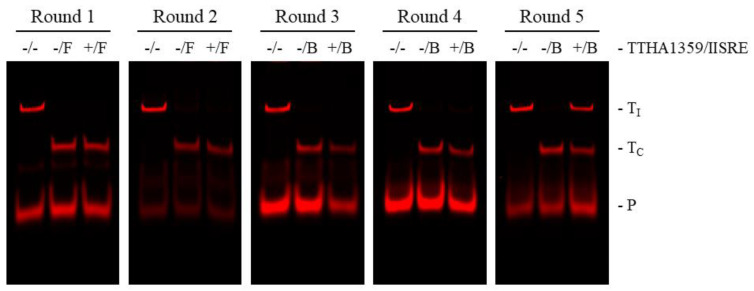
REPSA selection of TTHA1359-binding ST2R24 template DNAs. Each IR fluorescence image depicts selection reaction results from individual rounds of REPSA. Reactions in each figure correspond to DNA controls prepared without TTHA1359 or IISRE (−/−), cleavage controls including only IISRE (−/F or −/B representing FokI and BpmI IISRE inclusion, respectively), and the selection reaction prepared with 34 nM TTHA1359 protein and IISRE (+/F or +/B). Band designations: T_I_, intact ST2R24 template DNAs; T_C_, cleaved ST2R24 template DNAs; P, remnant 5′-labeled fluorescent ST2R primer.

REPSA selections were initiated with approximately 60 billion molecules of IRD7-labeled ST2R24 selection template and 34 nM purified TTHA1359 protein. Such provided a good representation of all possible 14-bp recognition sequences (4^14^/2 ~134 million) with the potential to identify sequences having nanomolar binding affinity. The first two rounds of REPSA were performed using the type IIS restriction endonuclease (IISRE) FokI, while the final three rounds used the IISRE BpmI. This was done to avoid selecting any intrinsically FokI cleavage-resistant DNAs, which were observed previously [25]. Nondenaturing PAGE analysis of 5′-fluorophore-labeled DNA species through the course of REPSA selection shows that a TTHA1359-dependent, IISRE cleavage-resistant species was first observed with Round 5 DNA and constituted over 50% of the product DNA (Figure 1). These data are consistent with the successful selection of DNAs containing high-affinity TTHA1359-binding sequences.

**Figure 2:**
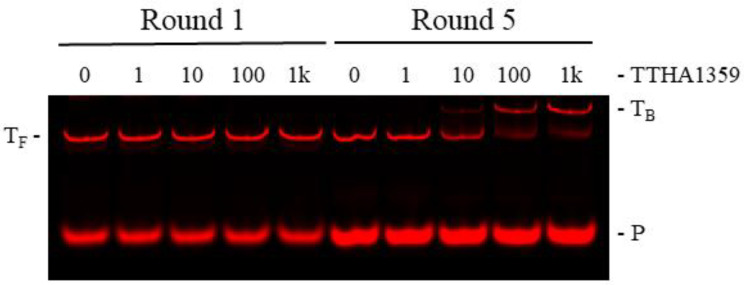
Validation of TTHA1359 REPSA-selected DNAs. PCR-amplified DNA (10 nM) from Round 1 (lanes 1–5) or Round 5 (lanes 6–10) REPSA selections were incubated with either 0, 1, 10, 100, or 1000 (1K) nM dimeric TTHA1359 protein. Band designations: T_F_, Free ST2R24 template DNAs; T_B_, bound ST2R24 template DNAs; P, remnant 5′-labeled fluorescent ST2R primer.

To validate REPSA selection of high-affinity TTHA1359-binding sequences, an independent protein-DNA binding assay, EMSA (electrophoretic mobility shift assay), was performed on the initial and final selected populations of DNA. DNAs from Round 5 demonstrated a shift to a single, slower mobility species for almost all input DNA following incubation with 100 nM dimeric TTHA1359, with the first indication of this species being observed at 10 nM dimeric TTHA1359 (Figure 2). No comparable effects were observed with Round 1 DNAs, even following incubation with 1000 nM dimeric TTHA1359. Taken together, these data indicate that REPSA was successful in selecting for DNAs that can form stable complexes with TTHA1359 and that a majority of our selected DNAs contained TTHA1359 binding sites.

### 2.2. Identification of Consensus TTHA1359-Binding Sequences

To determine consensus DNA-binding sequences for TTHA1359, REPSA Round 5 DNA was sequenced. Massively parallel semiconductor sequencing of a synthesized amplicon library yielded 9,516,545 total base reads with an incorrect base calling quality score, ≥Q20, of 8,631,131 and 158,313 reads of 60 bp average length. Sequencing1.java refinement reduced this to 61,754 sequences. Duplicates constituted less than 0.5% of the total sequences and were removed. Total and refined sequence data sets are available as Supplementary Materials (Data S1 and Data S2, respectively).

**Figure 3:**
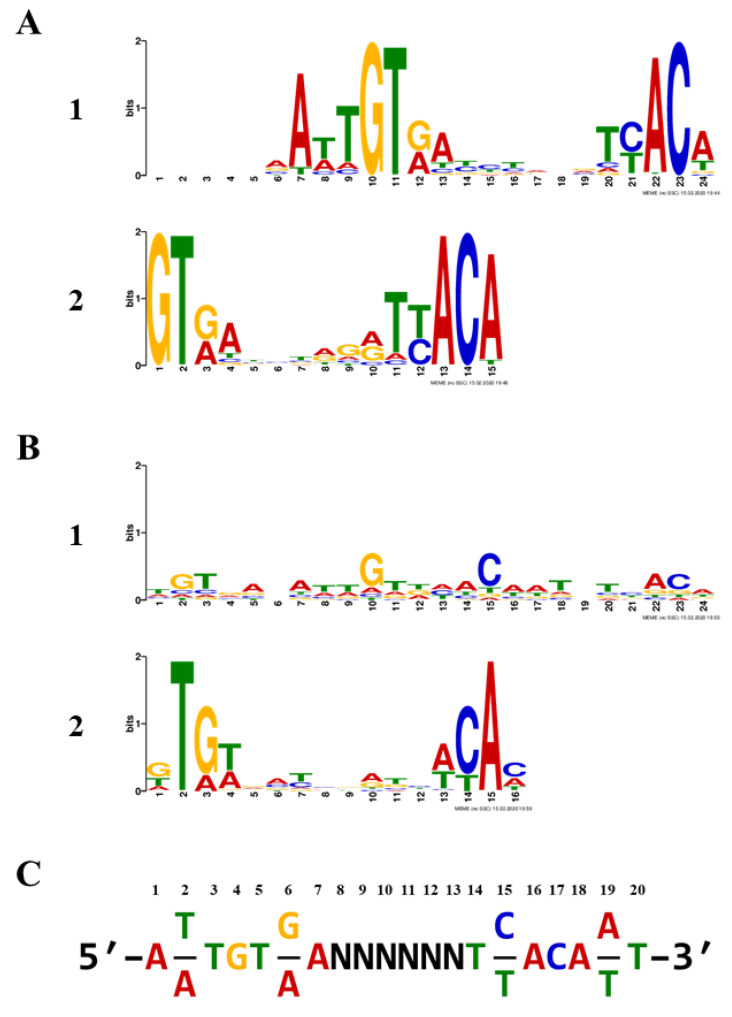
TTHA1359-binding motifs. Sequence logos were determined using MEME software with an input of 1000 Round 5 DNA sequences. (**A**) Top two non-palindromic motifs. (**B**) Top two palindromic motifs. (**C**) TTHA1359-DNA binding consensus sequence derived from MEME discovered motifs.

Sets of 1000 refined sequences were submitted to MEME analysis [26], both with and without palindromic filtering. The top non-palindromic motif was a 19-nt sequence within a 24-nt span and was present in 789/1000 input sequences for a statistical significance E-value of 1.1 × 10^−2996^. The top palindromic motif was 24-nt, found in 810/1000, and had an E-value of 4.5 × 10^−1153^. Both are rendered as sequence logos (Figure 3). Notably, the second-best motifs for each analysis, a 15-nt non-palindromic and a 16-nt palindromic motif, had significantly reduced E-values (4.2 × 10^−264^ and 1.5 × 10^−51^, respectively), in part due to their reduced lengths. We derived a 20-bp inverted repeat, primarily derived from the top non-palindromic motif, to serve as the TTHA1359-DNA binding consensus. This may be thought of as two palindromic 7-bp recognition elements separated by a 6-bp spacer region and is shown in Figure 3C. Such a motif would be expected for a CRP-family protein, which typically binds spaced, inverted repeat sequences as homodimers [27].

### 2.3. Biophysical Characterization of TTHA1359-DNA Binding

**Figure 4:**
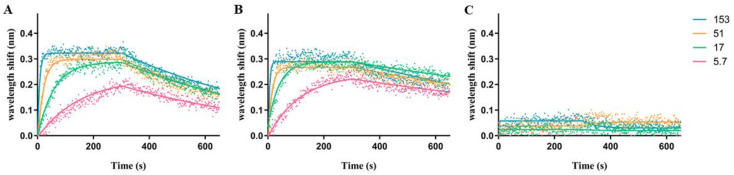
Representative BLI association and dissociation data plots. Graphs depict raw association and dissociation step data measured during BLI experiments with (**A**) ST2_1359_wt DNA, the wild-type TTHA1359 consensus sequence; (**B**) ST2_CRP_Ec DNA, the *E. coli* CRP consensus sequence; and (**C**) ST2_1359_ctrl DNA, the REPSAis control sequence. Dots depict raw data points. Solid lines depict calculated best-fit lines for raw data points. Line colors pink, green, orange, and cyan correspond to 5.7, 17, 51, and 153 nM dimeric TTHA1359, respectively.

Biolayer interferometry (BLI) assays, which ascertain binding kinetics in real-time by measuring the optical interference pattern of reflected white light upon macromolecular interaction with a biosensor, were performed to characterize TTHA1359-DNA binding interactions [28]. Raw BLI data (dots) for a range of TTHA1359 concentrations interacting with consensus or control sequences are shown in Figure 4. Nonlinear regression analysis of these data yielded the best-fitted association and dissociation curves (solid lines). From these, kinetic parameters, including association (*k*_on_) and dissociation (*k*_off_) rates, were derived. These, as well as dissociation constants and R^2^ coefficient of determination, were determined (Table 1). We also utilized BLI to test TTHA1359 binding to point mutations (Table 1; wt_p*) and insertion/deletion mutants within the spacer region (Table 1; wt_s*) of our REPSA-identified consensus sequence. Several of the mutant consensus sequences and a neutral control sequence, REPSAis (ctl), could not have their binding parameters determined under our experimental conditions, ostensibly given their weak binding by TTHA1359 (i.e., *K*_D_ > 1000 nM). We found that TTHA1359 bound its consensus sequence with high affinity (3.447 nM), in line with DNA binding by other CRP proteins [6]. Single point mutations of this sequence in just one of the 7-bp recognition elements reduced binding by tenfold or more, depending on the location of the mutation within the consensus, and primarily mirrored each base’s significance as ascertained by MEME. Interestingly, TTHA1359-DNA binding did not tolerate alterations in spacing between recognition elements, with either a single deletion (5-bp spacer) or addition (7-bp spacer) eliminating observable binding. Finally, TTHA1359 bound the CRP_EC_ consensus sequence with higher affinity than the REPSA identified consensus sequence. This may be the consequence of sequence differences on the CRP_EC_ consensus periphery (Table S1, compare sequences ST2_1359_wt and ST2_CRP_Ec) or the presence of an alternating AC spacer region in the TTHA1359 consensus.

### 2.4. Exploration of Potential Regulatory TTHA1359-DNA Binding Sites in the T. thermophilus HB8 Genome

**Figure 5:**
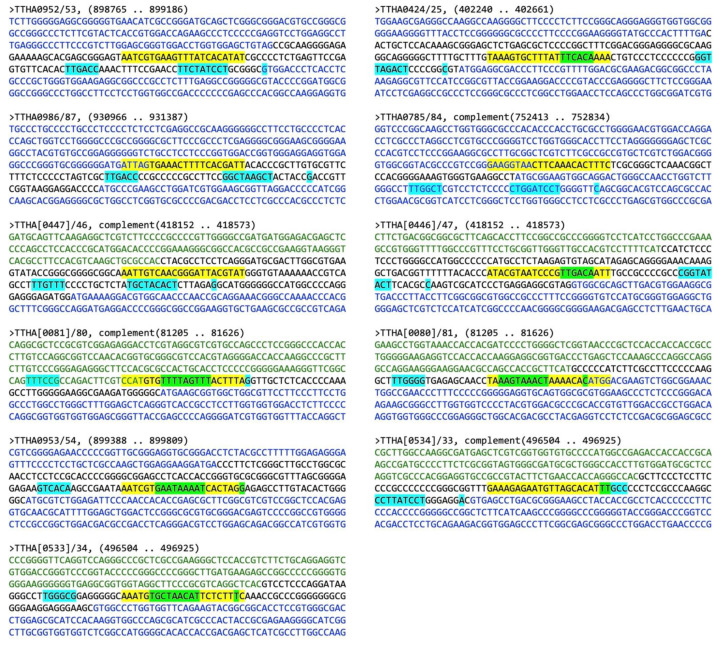
Predictions of TTHA1359-regulated *T. thermophilus* HB8 promoters. Shown are −200/+188 nucleotide sequences surrounding the FIMO-identified TTHA1359 binding site (Table 2). Names indicate the pairs of ORFs shown. Default is with a rightward, downstream orientation and is indicated with blue nucleotides. Reverse orientation genes have their names in brackets and are indicated with green nucleotides. Black nucleotides indicate intergenic regions. Potential core promoter elements (−35 and −10 boxes, +1 start site of transcription) were predicted using Softberry BPROM [30] and are indicated with cyan highlighting; TTHA1359-binding sites are indicated with yellow highlighting; overlapping TTHA1359-binding and core promoter elements are indicated by green highlighting.

The motif scanning program FIMO (Find Identified Motif Occurances) [29] was used to identify possible TTHA1359-binding sites within the *T. thermophilus* HB8 genome. Since the top non-palindromic motif discovered by MEME was a truncation of the consensus determined for TTHA1359, it was not directly imported into FIMO as previously described [21]. Instead, position-dependent letter-probability matrix data from positions 6–16 of the top non-palindromic motif were initially utilized to derive an extended 22-bp position-dependent letter-probability matrix. A text file suitable for FIMO upload and utilization was then written in MEME minimal motif format, containing the targeted version number of MEME, the extended motif alphabet and strand information, and the extended motif position-dependent letter-probability matrix (http://meme-suite.org/doc/meme-format.html?man_type=web [accessed on 13 February 2020]). The file was uploaded to FIMO v 5.0.5 and used to scan the GenBank *Thermus thermophilus* HB8 universal identifier 13,202 version 210 database for potential binding sites with statistically significant *p*-values less than 0.0001. The potential binding sites selected for further bioinformatic analysis were limited to those with *p*-values less than 5 × 10^−6^. These were examined for their positions relative to mapped open reading frames (ORFs) in the *T. thermophilus* HB8 genome. Those in intergenic regions or within the −200 to +20 nucleotide region most common for transcription activator binding were subjected to BPROM identification of potential promoter elements. Examples of these analyses, corresponding to FIMO binding sites with *p*-values < 5 × 10^−6^ and located in likely transcription regulatory regions, are shown in Table 2 and Figure 5, respectively.

Our promoter analyses found that 11 of the top 17 TTHA1359 genomic binding sites are situated in regions (intergenic, −200/+10) where bacterial transcriptional regulators typically reside. Five TTHA1359-binding sites were present in single, unidirectional promoters; three were shared by six opposing, bidirectional promoters. In each case, core promoter elements could be identified. Examples of different relationships between the TTHA1359 binding site and proximal core promoter elements were observed. Several had TTHA1359 sites upstream of the core promoter elements (e.g., *TTHA0953*, *TTHA0987*, *TTHA0784*, and *TTHA0446*). However, most had TTHA1359 sites overlapping core promoter elements, either the −35 box (e.g., *TTHA0425*, *TTHA0447*, and *TTHA0533*) or the −10 box (e.g., *TTHA0080*, *TTHA0081*, *TTHA0954*, and *TTHA0534*).

### 2.5. In Vitro Validation of TTHA1359-DNA Binding Sites in the T. thermophilus HB8 Genome

To validate TTHA1359-binding to FIMO-predicted gene promoters, we utilized the IISRE cleavage-protection assay REPA (restriction endonuclease protection assay) [31]. This assay is similar to REPSA; however, REPA uses defined DNA templates and excludes amplification and sequencing steps. We initially screened several promoter sequences and identified five FIMO-predicted promoter sequences that were resistant to IISRE cleavage in the presence of TTHA1359 compared to the REPSAis control probe (Figure S1). Notably, the two promoter sequences that showed little to no evidence of cleavage protection (*TTHA0954* and *TTHA0533/4*) had mutations in bases that we deemed essential based on our BLI analysis (Table 1).

**Figure 6:**
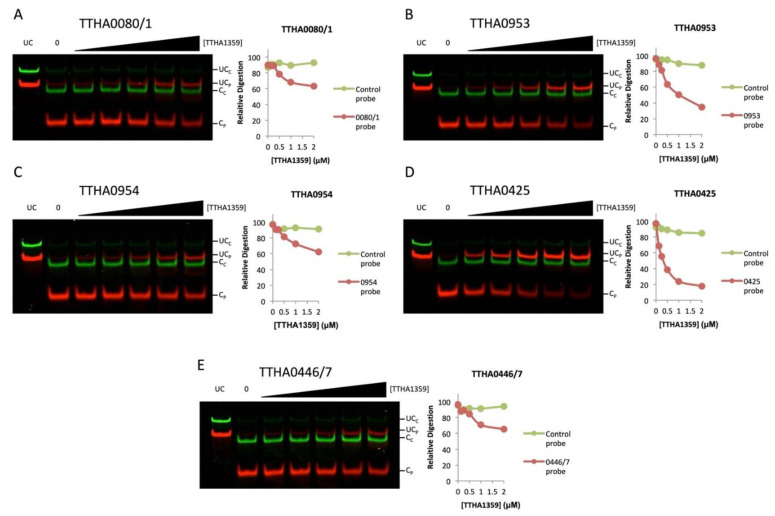
Validation of TTHA1359-regulated *T. thermophilus* HB8 promoters by REPA. DNA probes containing either a promoter region of the designated gene (red) or the REPSAis control sequence (green) were pre-incubated with increasing amounts of TTHA1359, ranging from 125 to 2000 nM monomeric protein (62.5 to 1000 nM dimeric protein), then subjected to restriction digest by the IISRE, BpmI. Levels of digestion for both DNA sequences were quantified and graphed. Band denotations: UC_C_, uncleaved control DNA; UC_P_, uncleaved promoter DNA; C_C_, cleaved control DNA; C_P_, cleaved promoter DNA. (**A**) *TTHA0080/1*, (**B**) *TTHA0953*, (**C**) *TTHA0954*, (**D**) *TTHA0425*, (**E**) *TTHA0446/7*.

We further analyzed the binding dynamics of TTHA1359 to the cleavage-resistant promoters by performing REPA with a titration of TTHA1359 (Figure 6). We observed levels of cleavage protection for each promoter sequence tested, with some exhibiting more protection than others (*TTHA0425* > *TTHA0953* > *TTHA0446/7*, *TTHA0954*, *TTHA0081*). Importantly, no observable cleavage protection was observed for the control REPSAis DNA (green) in each case. Collectively, these results show that TTHA1359 is capable of binding these promoters and further validate our reverse genetic and bioinformatical approach.

### 2.6. Bioinformatic Analysis of Potential TTHA1359-Regulated Genes

Further insights into the biological function of TTHA1359 as a transcriptional regulator were pursued through different bioinformatic approaches. For those FIMO-identified promoters potentially regulated by TTHA1359 that we validated in vitro, additional genes that could be part of a co-regulated operon were identified through BioCyc [32]. The resulting gene products and their biological roles were ascertained from information in the KEGG database [33]. Additionally, their gene expression changes between exponentially growing wild-type and isogenic TTHA1359-depleted strains were determined using GEO2R software and publicly available microarray data (GEO subseries GSE10369) [34]. These data are presented in Table 3. Some potential TTHA1359-regulated genes were involved in universal processes, including transcription (*TTHA0953*) and translation (*TTHA0446*, *tRNA-Ala-3*, and *TTHA0083*). Notably, those genes involved in translation were present in different transcriptional units (operons), suggestive of their coordinate regulation by TTHA1359. Others were involved in metabolic processes, including energy-related (*TTHA0425*) and sugar metabolism (*TTHA0954* and *TTHA0955*). A single operon (*TTHA0447–TTHA0451*) containing transporter genes thought to be involved with quorum sensing was also identified. Finally, several genes (*TTHA0080*, *TTHA0081*, and *TTHA0082*) lacked substantial information regarding their gene products and biological roles. This is understandable, given that over 40% of the genes in *T. thermophilus* HB8 encode hypothetical proteins with unknown biological functions [33].

## 3. Discussion

Using the iterative selection method REPSA, massively parallel sequencing, and MEME motif discovery software, we defined a 20-bp consensus sequence for TTHA1359, 5′–AWTGTRA(N)_6_TYACAWT–3′. This consensus contains a spaced inverted repeat, characteristic of most CRP-family transcription factor binding sites [27]. In fact, it is quite reminiscent of the archetype *E. coli* CRP consensus sequence ${5\prime -\mathrm{TGTGA}(N)}_{6}\mathrm{TCACA}-3\prime$ [6]. This is somewhat surprising, as although both proteins have recognizable CRP-type HTH domains (TTHA1359: aa 117–189, CRP_EC_: aa 138–210), there is not an appreciable identity or homology between these domains, except in the region conferring sequence-specific DNA recognition (TTHA1359: VRETVTK, CRP_EC_: SRETVGR, pfam13545: TRETVSR). Differences between these HTH domains may be necessary to maintain structural integrity under different environmental conditions, thermophilic and mesothermic, respectively.

Biophysical characterization of TTHA1359-DNA binding found that TTHA1359 binds its consensus sequence with a dissociation constant of 3.4 nM. Single point mutation of this consensus resulted in a decrease in binding affinity, from 6.8-fold to greater than 1000-fold, the measurement limit of our standard assay. The locations of the most critical nucleotides in the consensus sequence, 5′-A(T/A)TGT(G/A)A(N)_6_T(C/T)ACA(A/T)T-3′ (underlined), correlated well with those emphasized in the MEME-derived sequence logo. Such speaks to the validity of our REPSA approach. Not fully appreciated is the magnitude of single point mutations on binding affinity. For example, the related *T. thermophilus* HB8 CRP-family protein, TTHB099, recognizes a similar, albeit smaller, consensus sequence, 5′-TGT(A/g)N(Y)_3_(R)_3_N(T/c)ACA-3′, with some of the same nucleotides being most critical for binding affinity (underlined) [25]. However, mutation of these sites had only a 6- to 15-fold decrease in binding affinity, far less than observed for comparable mutations in TTHA1359 binding sites. Thus, our findings suggest that structurally similar proteins can bind related consensus sequences yet exhibit different responses to specific point mutations. They also suggest that slight changes in the DNA sequence that certain motif scanning algorithms may tolerate (e.g., FIMO) can profoundly affect the potential for TTHA1359 binding under concentration-limiting conditions. This is most evident by our in vitro analysis of FIMO-predicted promoter sequences, in which point mutations at critical bases based on our consensus sequence resulted in little to no TTHA1359 binding. Interestingly, although the initial adenine in our consensus sequence is essential for TTHA1359 binding, its palindromic thymine appears to be less critical (Figure S1C). This is consistent with previous reports suggesting one side of the TTHA1359 palindromic sequence may be more selective than the other [18,20].

Like most CRP-family proteins, TTHA1359 preferentially binds a spaced, inverted-motif sequence as a homodimer. However, we found that the length of this spacer region is critical. TTHA1359, like TTHB099 and CRP_EC_, binds to a core inverted repeat, 5′-GT(X)_10_AC-3′, with 10 intervening base pairs. This spacing allows both CRP-family homodimer members to be on the same face of the DNA double helix. In the case of TTHA1359, changes in this spacing, either shortening or lengthening by single base pair, result in a greater than a 100-fold decrease in binding. Curiously, similar spacing consequences are not usually observed for CRP_EC_ [36]. This demonstrates the unique importance of the spacing parameter on high-affinity TTHA1359-DNA binding.

To gain insights into the transcriptional functions of TTHA1359 in vivo, a GEO2R comparison between available microarray expression data for wild-type and isogenic TTHA1359-depleted *T. thermophilus* HB8 strains was performed. Expression changes in the genes immediately downstream of the FIMO-identified TTHA1359-binding sites, as well as other members of their transcriptional units (operons), were examined. Notably, only three of these genes (*TTHA0425*, *TTHA0081*, and *TTHA0082*) were among the top 100 GEO2R-identified responsive genes, while another (*TTHA0953*) was at position #102. Most of the remaining FIMO-identified genes were ranked much higher, either because of their low magnitude of expression change between wild-type and depleted strains, the low confidence in the results between multiple experiments, or both. While it is somewhat reassuring that our FIMO-identified sites with the highest correlation to the consensus TTHA1359 sequence were among the best GEO2R-identified TTHA1359-responsive genes, it is not wholly unexpected that a complete correlation between the two data sets was not observed. Transcriptional regulation in vivo is complex, relying on multiple proteins and co-factors. It can also be a consequence of indirect effects, e.g., regulation of transcriptional regulatory proteins beyond the one under investigation. Thus, while simple transcriptional repressors (e.g., *TTHA0101*, *TTHA0973*, and *TTHB023*) have shown a high degree of correlation between their FIMO-identified promoters and those genes exhibiting substantially increased expression in the depleted strains [22,23,24], we have found that putative dual-function transcriptional regulatory proteins (e.g., TTHB099) do not always exhibit such a direct relationship [25].

TTHA1359, also known as SdrP, has been investigated previously [18,20]. Using either changes in gene expression between wild-type and TTHA1359-depleted *T. thermophilus* HB8 strains or microarray data from 117 different environmental and chemical stress conditions, the authors were able to identify 16 gene promoters whose regulation by TTHA1359 could be recapitulated in an in vitro transcription assay. Analysis of their promoter regions allowed a refinement of the TTHA1359 consensus sequence to 5′-TTGTG(N)_9_CNC-3′, with these sites being located adjacent or overlapping the −35 box. Taken together, their data suggests that SdrP likely functions as a Class II transcriptional activator to primarily regulate gene expression in response to oxidative stress.

Notably, only one gene, *TTHA0425*, was shared between those identified through our REPSA-based, reverse genetic approach and those identified through the more conventional genetic process. Such differences may reflect the intrinsic limitations and biases of the assays used, REPSA/REPA versus microarrays/in vitro transcription. However, it is intriguing that none of these TTHA1359 binding sites would be considered high affinity based on their sequences. Perhaps under oxidative stress conditions, TTHA1359 accumulates to micromolar concentrations, thereby activating its target genes with weak promoter binding sites. This increase in cellular protein concentration provides a reasonable model for gene regulation by TTHA1359, especially as it lacks a modulatory co-factor like 3′−5′ cAMP, which many CRP family transcription factors require.

## 4. Materials and Methods

### 4.1. Oligonucleotides

Single-stranded oligodeoxyribonucleotides used in this study (Supplementary Table S1) were obtained from Integrated DNA Technologies (Coralville, IA, USA). Initial and subsequent selected ST2R24 REPSA selection libraries were prepared by PCR using unmodified ST2L and 5′-IRDye^®^ 700-modified IRD7_ST2R primers, essentially as previously described [21]. These libraries contained ST2R24 selection templates and 73-bp double-stranded deoxyribonucleotides with a central randomized 24-bp core flanked by defined sequences possessing IISRE-binding sites for FokI and BpmI [21]. Their design permitted the probing of sequence-specific protein-DNA binding through their inhibition of IISRE cleavage within the randomized core and the survival of intact templates for subsequent PCR amplification [37]. Defined, duplex DNA probes for biolayer interferometry (BLI) analyses were synthesized by PCR using ST2Ls and 5′-biotin-modified IRD7-ST2R primers, as previously described [21]. 5′-modified probe concentrations were measured with a Qubit 3 Fluorometer using our standard protocol [38].

### 4.2. TTHA1359 Protein

Purified full-length (1–202 aa) TTHA1359 protein was obtained from IPTG-induced *E. coli* BL21(DE3) bacteria transformed with the pET11a-derived expression plasmid PC011359-41 (RIKEN Bioresource Research Center) following heat-treatment of soluble bacterial extracts as previously described [24]. Coomassie-stained SDS-PAGE analysis of fractions through purification is shown in Supplementary Figure S2. Stock TTHA1359 is estimated to be 110 μM and greater than 95% pure.

### 4.3. TTHA1359 Consensus Sequence Determination

REPSA selections with 34 nM TTHA1359 were performed essentially as previously described [21]. Rounds 1–2 used 0.8 U type II restriction endonuclease (IISRE) FokI, while Rounds 3–5 had 2 U IISRE BpmI. Following each REPSA round, DNA products were assayed by native PAGE and IR fluorescence imaging following our standard protocol [39]. REPSA selections were concluded once a TTHA1359-dependent, IISRE cleavage-resistant population was observed.

The amplicon library preparation, Ion PGM individual sequencing particle preparation, Ion PGM semiconductor sequencing, and Ion Torrent sever sequence processing were all performed as previously described [21]. The resulting fastq raw sequences (Supplementary Data S1) were processed using our Sequencing1.java program and DuplicatesFinder v 1.1 to yield data (Supplementary Data S2) suitable for consensus sequence determination using Multiple Em for Motif Elicitation (MEME) v 5.0.5 (http://meme-suite.org/tools/meme [accessed on 13 February 2020]) [26]. MEME analysis was performed using default parameters, with and without a palindromic filter, and results were obtained as a position-weight matrix represented as a sequence logo.

### 4.4. Protein-DNA Binding Assays

Electrophoretic mobility shift assays (EMSA) were performed with REPSA-selected DNA populations, as previously described [21]. Biolayer interferometry (BLI) was performed using biotin-labeled DNA probes immobilized onto streptavidin biosensor tips (FortéBio) as previously described [24]. Five concentrations of TTHA1359 (5.7, 17, 51, 153, and 461 nM) were used to characterize each DNA probe. Global values for *k*_on_ and *k*_off_ rate constants and derived K_D_ equilibrium binding constants were determined from the BLI data using nonlinear regression analysis Association then Dissociation (GraphPad Prism v 5.03). R^2^ goodness-of-fit determinations were >0.95 in almost all cases. Restriction endonuclease digestion assays (REPA) were performed similarly as described previously [31]. Briefly, 1 nM of each 63-bp IR7-labeled promoter DNA sequence (red) and 86-bp IR8-labeled control DNA sequence (REPSAis; green) were incubated with increasing concentrations of TTHA1359 for 20 min at 55 °C. An amount of 0.8 U of the IISRE, BpmI, was added to each reaction and incubated for 5 min at 55 °C. Reactions were stopped by the addition of Orange Loading Dye (New England Biolabs) and 0.2% SDS. Samples were separated by native 10% PAGE and visualized using a LI-COR Odyssey Imager.

### 4.5. Bioinformatic Determination of Candidate Regulated Genes

Potential TTHA1359 binding sites within the *T. thermophilus* HB8 genome were identified using Find Identified Motif Occurrences (FIMO) v 5.0.5 (http://meme-suite.org/tools/fimo [accessed on 17 February 2020]) [29], with extended 22-nucleotide position-weight matrices derived from MEME and the GenBank Bacteria Genomes and Proteins *Thermus thermophilus* HB8 uid13202 v 210 serving as inputs. Stringency was limited to include only those matches having *p*-values ≤ 6.00 × 10^−5^. Sequences −210/+310 nucleotides were obtained from the Kyoto Encyclopedia of Genes and Genomes (KEGG) [33] *T. thermophilus* HB8 database (https://www.genome.jp/kegg-bin/show_organism?org=T00220 accessed on 28 February 2020) and the neighboring genes were identified. Each was scanned using Softberry BPROM (http://www.softberry.com accessed on 28 February 2020) [30] to identify potential bacterial core promoter elements. Those suggestive of TTHA1359 regulation had their transcription unit information, e.g., operon membership, ascertained through the *Thermus thermophilus* HB8 reference genome (https://biocyc.org/organism-summary?object=GCF_000091545 accessed on 28 February 2020), which is a part of the BioCyc database collection [32]. Postulated biological functions of TTHA1359-regulated genes were obtained from the definition or KEGG Orthology fields of the KEGG database. Changes in gene expression between wild-type and TTHA1359-deficient *T. thermophilus HB8* were obtained using the NCBI GEO2R program and series GSE10369 datasets from the NCBI GEO website (https://www.ncbi.nlm.nih.gov/geo/ [accessed on 28 February 2020]) [34]. Both changes in gene expression (LogFC values) and their statistical significance (*p*-values) were determined.

## Figures and Tables

**Table 1:** TTHA1359-DNA binding parameters.

Name	Sequence	$k_{\mathrm{on}}{\;(M}^{-1}\cdot s^{-1})$	$k_{\mathrm{off}}{\;(s}^{-1})$	$K_{D}{\;(\mathrm{nM})}_{\;}$	R^2^
wt	ATTGTGACACACATCACAAT	457,555	0.001577	3.447	0.9572
wt_p1	cTTGTGACACACATCACAAT	⸺⸺⸺⸺⸺⸺ Ambiguous ⸺⸺⸺⸺⸺⸺			
wt_p2	AgTGTGACACACATCACAAT	317,173	0.01166	36.78	0.9820
wt_p3	ATgGTGACACACATCACAAT	215,769	0.01867	86.53	0.9793
wt_p4	ATTtTGACACACATCACAAT	⸺⸺⸺⸺⸺⸺ Ambiguous ⸺⸺⸺⸺⸺⸺			
wt_p5	ATTGgGACACACATCACAAT	⸺⸺⸺⸺⸺⸺ Ambiguous ⸺⸺⸺⸺⸺⸺			
wt_p6	ATTGTtACACACATCACAAT	288,341	0.02153	74.67	0.9766
wt_p7	ATTGTGgCACACATCACAAT	267,033	0.006236	23.35	0.9843
wt_s5	ATTGTGAcacacTCACAAT	⸺⸺⸺⸺⸺⸺ Ambiguous ⸺⸺⸺⸺⸺⸺			
wt_s7	ATTGTGAcacacacTCACAAT	⸺⸺⸺⸺⸺⸺ Ambiguous ⸺⸺⸺⸺⸺⸺			
CRP_Ec	AAATGTGATCTAGATCACATTT	726,387	0.0007341	1.011	0.8979
ctrl	ATACGAAAAACACACAC	⸺⸺⸺⸺⸺⸺ Ambiguous ⸺⸺⸺⸺⸺⸺			

(Sequence) Lowercase nucleotides indicate a mutation from the TTHA1359 consensus sequence (wt). (CRP_Ec) Consensus DNA-binding sequence for CRP_EC_ [6]. (Ambiguous) Kinetic parameters could not be determined conclusively for the concentrations of TTHA1359 investigated. For these DNAs, estimated *K*_D_ > 1000 nM.

**Table 2:** TTHA1359-consensus sequences mapped in the genome of *T. thermophilus* HB8.

Start	End	*p*-Value	*Q*-Value	Sequence	Loc	Gene	Op
917,761	917,782	1.01 × 10^−^^8^	0.0125	AAATGTGAACATATTCACTTTC	−376	*TTHA0973*	1/6
898,965	898,986	1.48 × 10^−^^8^	0.0125	AATCGTGAAGTTTATCACATAT	−64	*TTHA0953*	S
1503	1524	1.78 × 10^−^^8^	0.0125	GAAAGTGAGATAACTCACATAT	+624	*TTHC002*	S
402,440	402,461	1.01 × 10^−^^8^	0.0532	TAAAGTGCTTTATTTCACAAAA	−34	*TTHA0425*	S
809,120	809,141	2.64 × 10^−7^	0.104	AATTGTGCTGGGCCACACAAAT	+975	*TTHA0843*	1/3
931,166	931,187	2.95 × 10^−7^	0.104	ATTAGTGAAACTTTTCACGATT	−95	*TTHA0987*	S
752,613	752,634	3.71 × 10^−7^	0.105	GAAGGTAACTTCAAACACTTTC	−44	*TTHA0784*	S
418,352	418,373	3.97 × 10^−7^	0.105	AATTGTCAACGGGATTACGTAT ATACGTAATCCCGTTGACAATT	−90 −54	*TTHA0446* *TTHA0447*	S 1/5
81,405	81,426	5.35 × 10^−7^	0.113	CCATGTGTTTTAGTTTACTTTA TAAAGTAAACTAAAACACATGG	−46 −18/+4	*TTHA0080* *TTHA0081*	1/2 1/3
899,588	899,609	5.82 × 10^−7^	0.113	AATCGTGAATAAAATCACTAGG	−22	*TTHA0954*	1/2
932,531	932,552	5.88 × 10^−7^	0.113	TCTTGTACTTTTATTCACGATT	+1250	*TTHA0987*	S
871,755	871,776	1.69 × 10^−6^	0.298	ACTTGTCAGCAAAATTACGATG	+620	*TTHA0911*	S
613,187	613,208	2.35 × 10^−6^	0.383	CAATGTCCTTTTAAGCTCAATT	+306	*TTHA0644*	2/3
496,704	496,725	3.11 × 10^−6^	0.471	GAAAGAGAATGTTAGCACATTT AAATGTGCTAACATTCTCTTTC	−36 −34	*TTHA0533* *TTHA0534*	1/2 1/2

(*p*-value) The probability of a random sequence of the same length matching the sequence’s position with an as good or better score. (*Q*-value) False discovery rate if the occurrence is accepted as significant. (Loc) Location of the TTHA1359-binding site relative to the start site of transcription. (Gene) Proximal gene downstream of the TTHA1359 binding sequence. (Op) Gene position within the postulated operon. (S) No operon, single transcriptional unit.

**Table 3:** Bioinformatic data for FIMO-identified, TTHA1359-regulated genes.

Operon	Gene	Product	Role	LogFC	Adj. *p*-Value
S	*TTHA0953*	UP (HTH MarR family)	Transcription	1.29	0.111
S	*TTHA0425*	NADH dehydrogenase (ubiquinone)	Energy metabolism	−1.82	0.00416
S	*TTHA0446*	50S ribosomal protein L34 (rpmH)	Translation	nd	nd
1	*TTHA0447*	Branched-chain amino acid transporter ATP-binding protein	Quorum sensing	−0.0356	0.972
2	*TTHA0448*	Long-chain-fatty-acid--CoA ligase	Quorum sensing	−0.461	0.684
3	*TTHA0449*	Branched-chain amino acid ABC transporter, permease protein	Quorum sensing	−1.18	0.412
4	*TTHA0450*	Branched-chain amino acid ABC transporter, permease protein	Quorum sensing	−0.417	0.780
5	*TTHA0451*	Branched-chain amino acid ABC transporter, amino acid-binding protein	Quorum sensing	−1.35	0.384
1	*TTHA0080*	UP	?	1.03	0.219
2	*tRNA-Ala-3*	tRNA^ala^	Translation	nd	nd
1	*TTHA0081*	UP (Rad52_Rad22-like)	?	0.998	0.100
2	*TTHA0082*	Metallophosphoesterase	?	1.00	0.107
3	*TTHA0083*	16S rRNA dimethyladenosine transferase	Translation	0.410	0.558
1	*TTHA0954*	Mannosyl-3-phosphoglycerate synthase	Sugar metabolism	0.530	0.306
2	*TTHA0955*	Mannosyl-3-phosphoglycerate phosphatase	Sugar metabolism	0.0925	0.865

(Operon) Numbers indicate positions of the genes within the operon. (S) Single transcriptional unit. (Product) Gene product identified in the KEGG database [33]. (UP) Uncharacterized protein. (Role) Biological function identified in the KEGG database [33]. (?) Unknown biological role. (LogFC) Log2-fold change between data obtained from TTHA1359-depleted and wild-type *T. thermophilus* HB8 strains, Series GSE10369. (Adj. *p*-value) The *p*-value was obtained following multiple testing corrections using the default Benjamini and Hochberg false discovery rate method [35]. (nd) Not determined.

## Data Availability

All data in this study are provided as Supplementary Materials or are publicly archived datasets. Links to these may be found in the *Materials and Methods*.

## Supplementary Materials

The following are available online at https://www.mdpi.com/article/10.3390/ijms221810042/s1.
