# Supplementary material for: Discovering the DNA-Binding Consensus of the Thermus thermophilus HB8 Transcriptional Regulator TTHA1359
Source: Int J Mol Sci. 2021 Sep 17;22(18):10042. doi: 10.3390/ijms221810042 (PMC8465061; doi:10.3390/ijms221810042)
Supplement: Supplementary file 1 [file ijms-22-10042-s001.zip › Figure S1.pdf]

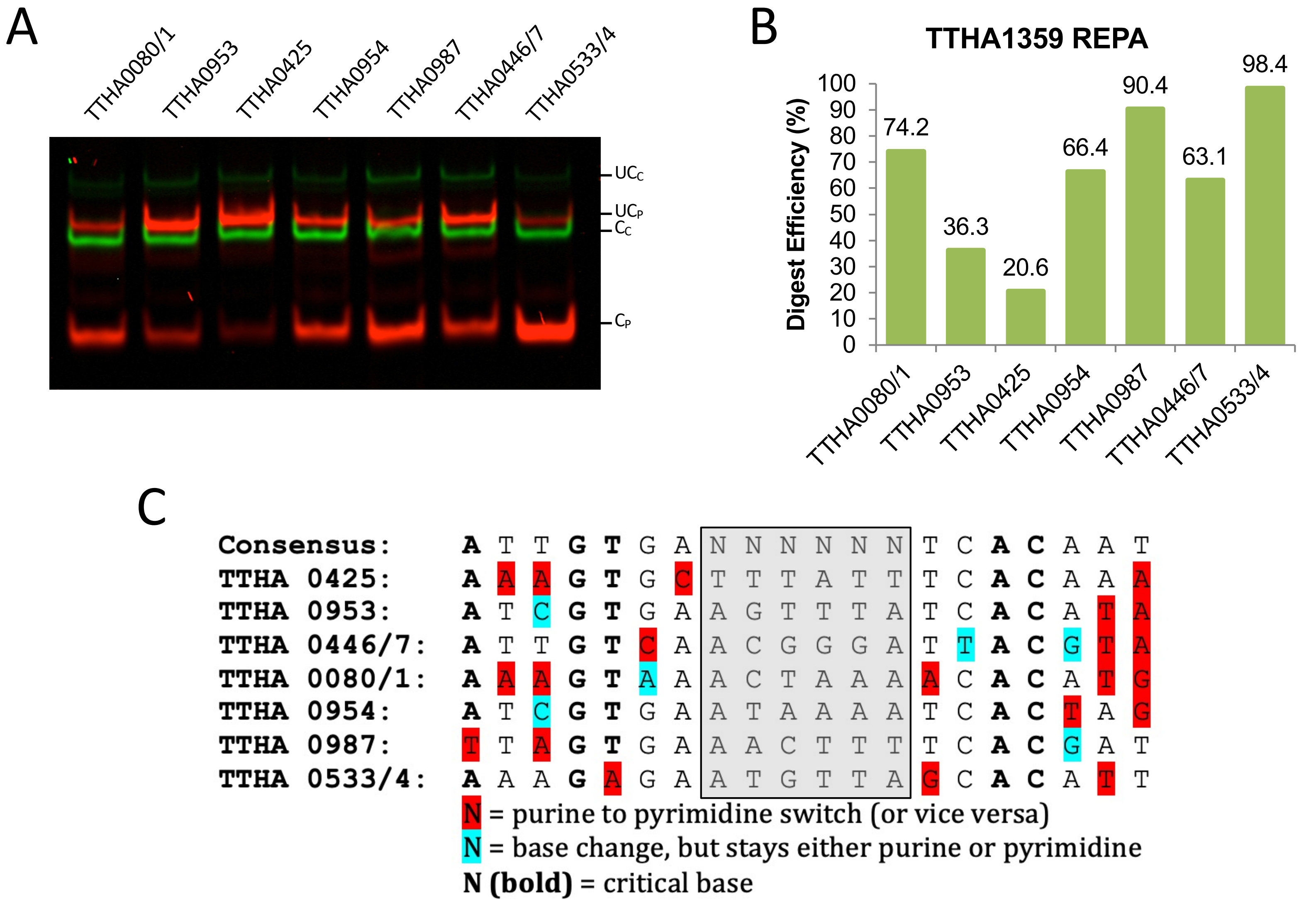

Figure S1. Screen of potential TTHA1359-regulated *T. thermophilus* HB8 promoters by REPA. (**A**) DNA probes containing either a promoter region of the designated gene (red) or the REPSAis control sequence (green) were pre-incubated with 1μM TTHA1359, then subject to restriction digest by the IISRE, BpmI. Band denotations: UC_C, uncleaved control DNA; UC_p, uncleaved promoter DNA; C_C, cleaved control DNA; C_P, cleaved promoter DNA. (**B**) Levels of digestion in (**A**) were normalized to the digestion efficiency of the control probe and graphed. (**C**) Sequence alignment of all promoter probes tested in (**A**) to the RESPA-identified consensus sequence. Deviations from the consensus sequence are highlighted and critical bases are in bold.
