# Supplementary material for: Discovering the DNA-Binding Consensus of the Thermus thermophilus HB8 Transcriptional Regulator TTHA1359
Source: Int J Mol Sci. 2021 Sep 17;22(18):10042. doi: 10.3390/ijms221810042 (PMC8465061; doi:10.3390/ijms221810042)
Supplement: Supplementary file 1 [file ijms-22-10042-s001.zip › Figure S2.pdf]

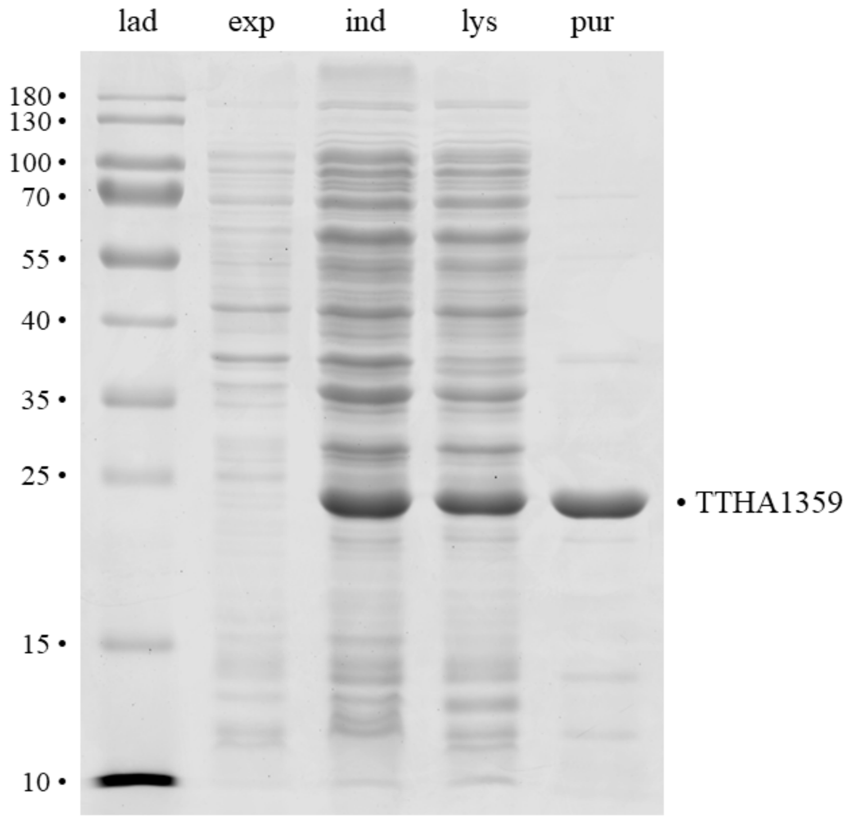

Figure S2. Expression and purification of TTHA1359 protein. The image depicts samples obtained during TTHA1359 protein expression and purification as analyzed by 12% SDS-PAGE. Lanes were loaded with protein ladder (lad), cultivated transformant *E. coli* in the exponential growth phase (exp), induced, cultivated transformant *E. coli* post-induction with 1 mM IPTG (ind), recovered cell lysate following harvested bacterial cell membrane disruption (lys), and recovered soluble cell content after high-temperature purification (pur). Molecular weights of the protein ladder are indicated to the left of the figure. The protein band corresponding to the TTHA1359 monomer is indicated to the right of the figure.
