# Supplementary material for: Discovering the DNA-Binding Consensus of the Thermus thermophilus HB8 Transcriptional Regulator TTHA1359
Source: Int J Mol Sci. 2021 Sep 17;22(18):10042. doi: 10.3390/ijms221810042 (PMC8465061; doi:10.3390/ijms221810042)
Supplement: Supplementary file 1 [file ijms-22-10042-s001.zip › Table S1.pdf]

Table S1. Oligonucleotides

Name	Sequence	Length	Purif.	Use
ST2R24	CTAGGAATTCGTGCAGAGGTGAATNNNNNNNNNNNNNNNNNNNNNNNNTTACCATCCCTCCAGAAGCTTGGAC	73	PAGE	ST2R24 Template Precursor
ST2L	CTAGGAATTCGTGCAGAGGTGAAT	24	Desalt	PCR Primer
ST2Ls	CTAGGAATTCGTGCAGAGGTGA	22	Desalt	PCR Primer
IRD7_ST2R	/5IRD700/GTCCAAGCTTCTGGAGGGATGGTAA	25	HPLC	5′-labeled IRDye700 PCR Primer
A_BC06_ST2R	CCATCTCATCCCTGCGTGTCTCCGACTCAGCTGCAAGTTCGATGTCCAAGCTTCTGGAGGGATG	64	PAGE	Fusion PCR Primer
trP1_ST2L	CCTCTCTATGGGCAGTCGGTGATCTAGGAATTCGTGCAGAGGTGA	45	PAGE	Fusion PCR Primer
A_uni	CCATCTCATCCCTGCGTG	18	Desalt	PCR Primer
trP1_uni	CCTCTCTATGGGCAGTCGG	19	Desalt	PCR primer
Bio_ST2R	/5BiodT/GTCCAAGCTTCTGGAGGGATG	22	HPLC	5′-biotinylated PCR primer
ST2_1359_ctrl	CTAGGAATTCGTGCAGAGGTGAATACGAAAAACACACACCATCCCTCCAGAAGCTTGGAC	60	Desalt	TTHA1359 control probe precursor
ST2_1359_wt	GGAATTCGTGCAGAGGTGAATTGTGACACACATCACAATCATCCCTCCAGAAGCTTGG	58	Desalt	TTHA1359 consensus probe precursor
ST2_1359_wt_p1	GGAATTCGTGCAGAGGTGACTTGTGACACACATCACAATCATCCCTCCAGAAGCTTGG	58	Desalt	TTHA1359 mutant position 1 consensus probe precursor
ST2_1359_wt_p2	GGAATTCGTGCAGAGGTGAAGTGTGACACACATCACAATCATCCCTCCAGAAGCTTGG	58	Desalt	TTHA1359 mutant position 2 consensus probe precursor
ST2_1359_wt_p3	GGAATTCGTGCAGAGGTGAATGGTGACACACATCACAATCATCCCTCCAGAAGCTTGG	58	Desalt	TTHA1359 mutant position 3 consensus probe precursor
ST2_1359_wt_p4	GGAATTCGTGCAGAGGTGAATTTTGACACACATCACAATCATCCCTCCAGAAGCTTGG	58	Desalt	TTHA1359 mutant position 4 consensus probe precursor
ST2_1359_wt_p5	GGAATTCGTGCAGAGGTGAATTGGGACACACATCACAATCATCCCTCCAGAAGCTTGG	58	Desalt	TTHA1359 mutant position 5 consensus probe precursor
ST2_1359_wt_p6	GGAATTCGTGCAGAGGTGAATTGTTACACACATCACAATCATCCCTCCAGAAGCTTGG	58	Desalt	TTHA1359 mutant position 6 consensus probe precursor
ST2_1359_wt_p7	GGAATTCGTGCAGAGGTGAATTGTGGCACACATCACAATCATCCCTCCAGAAGCTTGG	58	Desalt	TTHA1359 mutant position 7 consensus probe precursor
ST2_1359_wt_s5	GGAATTCGTGCAGAGGTGAATTGTGACACACTCACAATCATCCCTCCAGAAGCTTGG	57	Desalt	TTHA1359 shortened mutant consensus probe precursor
ST2_1359_wt_s7	GGAATTCGTGCAGAGGTGAATTGTGACACACACTCACAATCATCCCTCCAGAAGCTTGG	59	Desalt	TTHA1359 lengthened mutant consensus probe precursor
ST2_CRP_Ec	GGAATTCGTGCAGAGGTGAAATGTGATCTAGATCACATTTCATCCCTCCAGAAGCTTGG	59	Desalt	*E. coli* CRP consensus probe precursor

Random oligonucleotides denoted by N. /5IRD700/ 5′-IRDye® 700 modification. /5BiodT/ 5′-biotin-C_6-deoxythymidine modification. Length reported in nucleotides.
